# VGLL3 expression is associated with macrophage infiltration and predicts poor prognosis in epithelial ovarian cancer

**DOI:** 10.3389/fonc.2023.1152991

**Published:** 2023-06-05

**Authors:** Razaul Haque, Jaebon Lee, Joon-Yong Chung, Ha-Yeon Shin, Hyosun Kim, Jae-Hoon Kim, Jae Won Yun, Eun-Suk Kang

**Affiliations:** ^1^ Department of Laboratory Medicine and Genetics, Samsung Medical Center, Seoul, Republic of Korea; ^2^ School of Medicine, Sungkyunkwan University, Suwon, Republic of Korea; ^3^ School of Medicine, Sungkyunkwan University, Seoul, Republic of Korea; ^4^ Molecular Imaging Branch, Center for Cancer Research, National Cancer Institute, National Institutes of Health, Bethesda, MD, United States; ^5^ Department of Obstetrics and Gynaecology, Gangnam Severance Hospital, Yonsei University College of Medicine, Seoul, Republic of Korea; ^6^ Veterans Medical Research Institute, Veterans Health Service Medical Center, Seoul, Republic of Korea; ^7^ Cell and Gene Therapy Institute, Research Institute for Future Medicine, Samsung Medical Center, Seoul, Republic of Korea

**Keywords:** high-grade serous ovarian carcinoma, hippo pathway, VGLL3, prognosis, macrophage

## Abstract

**Background/objective:**

High-grade serous ovarian carcinoma (HGSOC) is the most common histologic type of epithelial ovarian cancer (EOC). Due to its poor survival outcomes, it is essential to identify novel biomarkers and therapeutic targets. The hippo pathway is crucial in various cancers, including gynaecological cancers. Herein, we examined the expression of the key genes of the hippo pathway and their relationship with clinicopathological significance, immune cells infiltration and the prognosis of HGSOC.

**Methods:**

The Cancer Genome Atlas (TCGA) and Gene Expression Omnibus (GEO) data were curated to analyse the mRNA expression as well as the clinicopathological association and correlation with immune cell infiltration in HGSOC. The protein levels of significant genes in the HGSOC tissue were analysed using Tissue Microarray (TMA)-based immunohistochemistry. Finally, DEGs pathway analysis was performed to find the signalling pathways associated with VGLL3.

**Results:**

VGLL3 mRNA expression was significantly correlated with both advanced tumor stage and poor overall survival (OS) (p=0.046 and p=0.003, respectively). The result of IHC analysis also supported the association of VGLL3 protein with poor OS. Further, VGLL3 expression was significantly associated with tumor infiltrating macrophages. VGLL3 expression and macrophages infiltration were both found to be independent prognostic factors (p=0.003 and p=0.024, respectively) for HGSOC. VGLL3 was associated with four known and three novel cancer-related signalling pathways, thus implying that VGLL3 is involved in the deregulation of many genes and pathways.

**Conclusion:**

Our study revealed that VGLL3 may play a distinct role in clinical outcomes and immune cell infiltration in patients with HGSOC and that it could potentially be a prognostic marker of EOC.

## Graphical Abstract

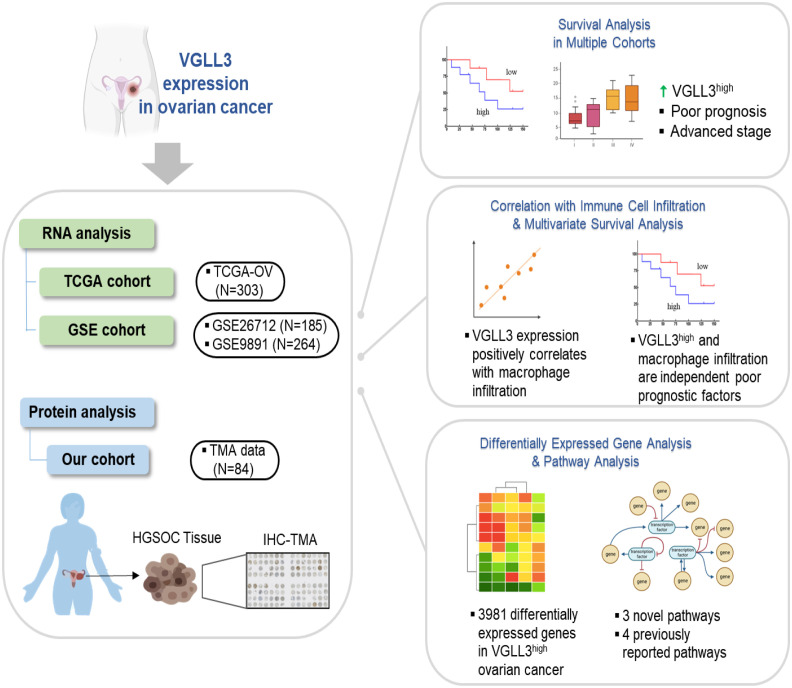

## Introduction

Epithelial ovarian cancer (EOC) is the most dominant form of ovarian cancer, which accounts for the highest rates of mortality and morbidity among the female sex (1). Despite the immense advancements in treatment strategy, the 5-year overall survival rate of EOC is still under 45% (2). Such poor prognosis may result from the complex and obscure pathogenesis of EOC, late diagnosis, a lack of predictive biomarkers, and ineffective target identification (3). Tumors in human ovary can be categorized into *surface epithelial-stromal* tumors, *sex cord-stromal* tumors, and *germ cell* tumors (4). Surface epithelial-stromal carcinoma can be further sub-grouped into serous (HGSOC and low-grade serous ovarian carcinoma), mucinous carcinoma, endometrioid carcinoma, clear cell carcinoma, and transitional cell carcinoma (or Brenner type (4). HGSOC considered as the most lethal EOC diagnosed at advanced stages, and results even higher percentage of mortality in ovarian cancer (5). However, characterization of HGSOC is very difficult, as they account for a very low number of mutations, and there is a scarcity of appropriate diagnosis markers. According to TCGA, the most common mutation found in Tp53 (96% cases), whereas mutations in other commonly mutated oncogenes such as *KRAS*, *BRAF*, *NRAS*, and *PIK3CA* are very rare in HGSOC (all less than 1%) (6, 7). Therefore, there is an urgent need to explore novel biomarkers to improve the diagnosis and early detection of HGSOC to improve treatment efficiency.

The hippo pathway is a critical regulator of morphogenesis, organ size determination, and tumorigenesis in many tissues, including the reproductive system (8, 9). Formation of the YAP/TAZ-TEADs complex serves as the key mechanism that stimulates the expression of target genes (e.g., connective tissue growth factor and cysteine-rich angiogenic inducer 61) that are essential for cell proliferation and survival (10). As a tumor suppressor pathway, dysregulation of the hippo pathway has been reported in various cancers (11). Yes-associated protein (YAP) and transcriptional coactivator with PDZ-binding motif (TAZ) plays an oncogenic role in EOC tumorigenesis by increasing cell proliferation and apoptotic resistance, reducing contact inhibition, and improving motility and anchorage-independent growth (12, 13). However, their clinicopathological outcomes in EOC are still debated.

Hippo pathway not only regulated by its core (YAP/TAZ) components, but also other regulatory and signalling molecules. Vestigial-like 3 (VGLL3) is a member of the VGLL family that also serves as a cofactor for transcriptional enhanced associate domains (TEADs) and participate in hippo signalling (14). TEAD1-4 recruits VGLL3 competitively to YAP/TAZ for their transcriptional activity (15). Despite the physiologic function is unknown, VGLL3 has recently been reported to be associated with the inhibition of adipocyte differentiation and the regulation of trigeminal nerve formation and cranial neural crest migration (16, 17). Now a day’s multiple pieces of evidence suggest that VGLL3 is associated with different cancers (18–21). VGLL3 has previously been reported to play a tumor suppressive role in EOC by Gambaro et al. in 2013 (22). Since then, there have been no further reports elucidating the role of VGLL3 in EOC. Moreover, Gambaro et al. derived the hypothesis from a chromosome transfer experiment wherein the transfer of a chromosome fragment containing *VGLL3* gene suppressed tumor phenotypes in the ovarian tumor cell line OV90 (23, 24). However, *VGLL3* as a single-gene transfer did not generate stable VGLL3 expression and was unable to suppress the proliferation of OV90 cells (22). Therefore, it is essential to re-evaluate the role of VGLL3 in EOC.

There is increasing evidence suggesting that immune cell infiltration plays a crucial role in the prognosis of various tumors and affects OS. Infiltration of different immune cells, such as T cells, macrophages, mast cells, and natural killer cells, is known to be associated with either favourable or unfavourable prognosis (25). Zhang et al. recently showed that *VGLL3* serves as a novel unfavourable prognostic biomarker in stomach adenocarcinoma and correlates with immune evasion, particularly due to infiltration of macrophages and dendritic cells (21). However, the role of VGLL3 in the immune microenvironment of EOC remains to be elucidated

In this study, we performed a comprehensive analysis using public databases and web tools, as well as tissue microarray (TMA) to investigate the significance of key genes in hippo pathways, including *VGLL3*, *VGLL4*, *TEAD3*, *TEAD4*, *YAP*, and *TAZ*, on the clinicopathological characteristics and immune cell infiltration features of HGSOC. We also investigated the role of VGLL3 as a prognostic factor of HGSOC and its association with cancer-related signal transduction pathways.

## Materials and methods

### mRNA data sources and clinical information

The information on the mRNA expression and the clinical data of ovarian cancer were acquired from the Cancer Genome Atlas (TCGA) repository, Genome Data Analysis Centre (GDAC) (https://gdac.broadinstitute.org/), and the University of California Santa Cruz browser (https://xenabrowser.net/datapages/). For further analysis, 303 samples that had clinical and mRNA information available were selected (**Table 1**). The expression of genes was compared between normal ovarian (n=88) and tumor (n=426) tissues based on the GEPIA2 database (26) (http://gepia2.cancer-pku.cn/#index). For the validation of gene expression in HGSOC, we acquired data from the gene expression omnibus (GEO) in the form of GSE26712 (10 normal ovarian *vs*. 185 tumor tissue) and GSE9891 (264 HGSOC tissue samples).

**Table 1:** Clinicopathological features of high-grade serous ovarian carcinoma patients from The Cancer Genome Atlas and TMA datasets.

Clinical Factor	TCGA (n=302)	TMA (n=84)
Age (years, mean ± SD)	59.10 ± 10.93	54.77 ± 10.41
CTx response (n) (Sensitive/Resistant/unknown)	200/40/62	67/15/2
Death/Alive (n)	183/119	50/34
Overall survival (years, mean ± SD)	3.04 ± 2.44	6.65 ± 5.03
Pre-CA125 (n) (Negative ≤35U/ml)/(Positive >35U/ml)	NA	7/77
Stage		
Stage I (n)	1	8
Stage II (n)	21	5
Stage III (n)	240	58
Stage IV (n)	38	13
Unknown	2	–
Grade		
G1 (n)	1	2
G2 (n)	33	38
G3 (n)	260	44
Others (GB, GX) (n)	8	–

(n), Number of patients; CR, Complete response; PR, Partial response; SD, Stable disease; PD, Progressive disease; CTx, Chemotherapy; Others (GB, GX), GB, Grade borderline; GX, Grade cannot be assessed; NA, not available.

### Cell culture

Four ovarian cancer cell lines, namely SKOV3, OVCAR3, OVCA429, OVCA433 and five primary EOC cell lines including YDOV-13 (originated from a malignant Brenner tumor), YDOV-139, YDOV-157, YDOV-161(originated from serous cystadenocarcinomas) and YDOV-151 (originated from a mucinous cystadenocarcinoma) were used in this study (27–30). The primary cell lines were established in Jae-Hoon Kim’s lab and all cell lines were kindly provided by Jae-Hoon Kim (Gangnam Severance Hospital, Yonsei University). SKOV3 and OVCAR3 cell lines were maintained in RPMI-1640 media supplemented with 10% FBS and 1% with penicillin/streptomycin. The other cell lines were maintained in DMEM media containing 10% FBS and 1% penicillin/streptomycin. All the cell lines were cultured at 37°C in 5% CO_2_.

### RNA isolation and real-time qPCR

At 70-80% of confluence, cells were washed with PBS, after which total RNA was extracted using TRIzol reagent (Ambion, Carlsbad, USA) according to the manufacturer’s protocol. Total RNA (1 μg) from each sample was reverse-transcribed into cDNA using the Maxima First Strand cDNA Synthesis Kit (Thermo Scientific, Waltham, MA) according to the manufacturer’s protocol. Real-time quantitative polymerase chain reaction (RT-qPCR) was performed to quantify mRNA expression using SYBR Green PCR Master Mix (Enzynomics, Daejeon, Republic of Korea) and the QuantStudio 6 Flex real-time PCR system (Applied Biosystems, Foster City, CA). Relative mRNA expression was quantified using the comparative Ct (ΔCt) method and expressed as 2^-ΔΔCt^. The following primers were used for PCR: VGLL3: Forward 5’- CCAACTACAGTCACCTCTGCTAC-3’ and Reverse 5’- ACCACGGTGATTCCTTACTCTTG-3’, GPADH: Forward 5’- ATGGAAATCCCATCACCATCTT-3’ and Reverse 5’- CGCCCCACTTGATTTTGG-3’.

### Protein extraction and western blotting

Total cell lysates were isolated using cell lysis buffer (RIPA buffer: Cell Signaling Technology #9806, Danvers, MA) containing protease inhibitor cocktail (Roche, Nutley, NJ). Protein concentrations were determined by BCA assay (Sigma-Aldrich, St. Louis, MO). Proteins were separated by SDS-PAGE and transferred from gels to 0.2 μm nitrocellulose membranes (Pall Corporation, Washington, NY). The nitrocellulose membrane was further incubated overnight at 4°C with rabbit anti-VGLL3 (1:1000, Novus Biologicals, NB100-56875, Centennial, USA) and rabbit anti-GAPDH (1:2000, Novus Biologicals, 4650S, Centennial, USA). After that membranes were incubated with HRP-conjugated anti-Rabbit IgG (1:1000, Cell Signaling Technology, 7074S, Danvers, MA) secondary antibody for 1 hour at RT, protein bands were visualised using western blotting luminol reagent (Santa Cruz Biotechology, Inc., Dallas, Texas).

### Patients’ tissue samples and clinical information

The unstained slides from 84 HGSOC and 66 adjacent normal ovarian epithelial TMA blocks and their corresponding sets of clinical information were obtained from the Korea Gynaecologic Cancer Bank (KGCB) of Gangnam Severance Hospital, Yonsei University College of Medicine (No. HTB-P2021-5), funded by the Korean Government Ministry of Science and ICT (MSIT) (NRF-2017M3A9B8069610). All the patients were treated with first line chemotherapy. Tissue samples and medical records were obtained with the approval of the Institutional Review Board of Gangnam Severance Hospital (IRB#, HTB-P2021-5), Seoul, Republic of Korea. All procedures were conducted in accordance with the guidelines of the Declaration of Helsinki. Tumor staging was performed according to the classification established by the International Federation of Gynaecology and Obstetrics (FIGO). For all study participants, CA125 levels were measured at primary diagnosis up to 1 week preoperatively using Elecsys CA125 II ECLIA (Roche Diagnostics, Rotkreuz, Switzerland). The demographics and clinical characteristics of the individuals that participated in this study are listed in **Table 1**.

### Tumor infiltrating immune cell estimation

The estimated abundance of tumor-infiltrating immune cells (TIICs) was calculated using immunedeconv in R, which was downloaded from the Tumor Immune Estimation Resource (TIMER) 2.0 website (http://timer.cistrome.org/) (31). To elaborate, TIICs were inferred using three different tools, including EPIC (http://epic.gfellerlab.org/), TIMER (https://cistrome.shinyapps.io/timer/), and CIBERSORT (https://cibersort.stanford.edu). These three computational algorithms use deconvolution-based approaches that model gene expressions as the weighted sum of the expression profiles of the admixed cell types (32–34). The outlier of TIICs was eliminated using Tukey’s method. Then, a correlation analysis between the abundance of TIICs and gene expressions was conducted using Pearson’s method. A *p*-value < 0.05 and a correlation co-efficient R ≥ 0.30 were considered to represent a significant correlation.

### Immunohistochemical analysis

VGLL3 protein expressions were analysed by immunohistochemical guided TMA, which was described before (35). Briefly, deparaffinised and rehydrated sections were retrieved *via* microwave for 10 min in a pH 6.0 citrate buffer. Endogenous peroxidase was then inactivated using peroxidase blocking solution (Agilent, S2023, Dako, Glostrup, Denmark) for 20 minutes. Next, the tissue samples were incubated with the anti-VGLL3 primary antibody (1:200, Novus Biologicals, NBP2-31590, Centennial, USA) for 2 h at 25°C. The secondary antibody was applied for 1 h at 25°C, after which detection was performed using DAB Substrate-Chromogen solution (Agilent, K5007, Dako, Glostrup, Denmark). Lastly, the sections were counterstained using haematoxylin and mounted.

Stained TMA slides were digitized using the NanoZomer XR digital pathology (NDP) system (Hamamatsu, Hamamatsu City, Japan) at ×40 objective magnification with a single-focus layer. Digitized images were automatically analysed using Visiopharm software version 6.9.1 (Visiopharm, Hørsholm, Denmark). Regarding the expression value of VGLL3 nuclear staining, a brown nuclear staining intensity (0=negative, 1=weak, 2=moderate, and 3=strong) and a respective percentage were obtained. For VGLL3 cytoplasmic assessment, a brown cytoplasmic intensity (weak and strong) was obtained, and each proportion was analysed. Histoscores were calculated by multiplying the percentage of positive cells by their staining intensity.

### Differentially expressed gene analysis

VGLL3 mRNA raw read counts downloaded from GDAC were used to identify the association with differentially expressed genes (DEGs) using the DESeq2 package in R, and the cut-off value of FDR (offered as adjusted p-value) was 0.001 (p-value < 2.04e-4) (36). Using the identified DEGs correlated with VGLL3, pathway analysis was performed using ConsensusPathDB (http://cpdb.molgen.mpg.de/) (37). Next, among the identified pathways (*p* value < 0.01, *q* value < 0.2), cancer-related pathways were selected through a literature review and manual curation. We also conducted a heatmap analysis using complex heatmap package in R to identify the significant genes that were associated with selected pathways and high VGLL3 expression.

### Statistical analysis

Data were statistically analysed using R software version 4.0.2. (R 4.0.2, Auckland, New Zealand). All data values are expressed as mean ± standard error of the mean (S.E.M). To compare gene expression among groups with different clinical and pathological features, the DESeq2 package, Mann-Whitney test, and Kruskal-Wallis test were used. For survival analysis, Kaplan-Meier plot and log-rank test were conducted using the survival and survminer packages in R (38). To identify the independent prognostic factor, Cox regression analyses were performed and visualised using the forest plot package in R (39). *p*-values <0.05 were considered to be statistically significant.

## Results

HGSOC tumors samples from 302 TCGA and 84 TMA datasets were analysed according to stage and grade. TMA data was further analysed based on age, pre-CA125 level, and chemosensitivity after initial treatment. CA125 levels of >35 IU/mL (n=77) were considered to be positive, while levels of ≤35 IU/mL (n=7) were considered to be negative.

### Altered *VGLL3* mRNA expression had a prognostic significance in HGSOC

**Figure 1:**
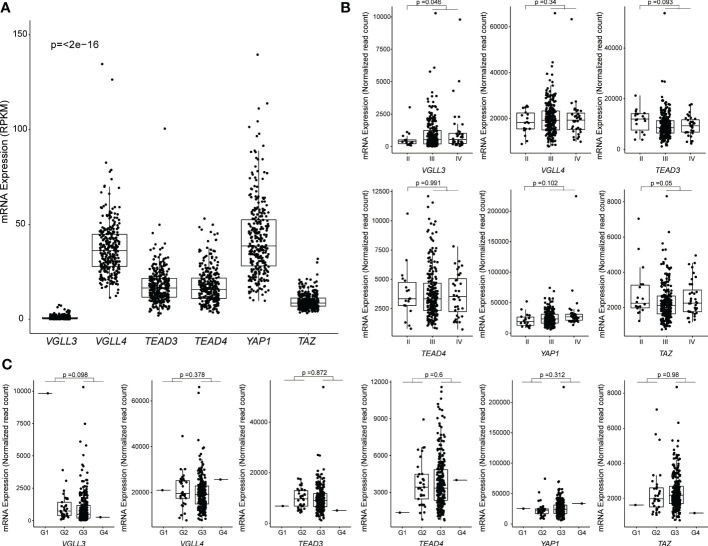
Correlation between mRNA expression and clinicopathological features in The Cancer Genome Atlas (TCGA) ovarian cancer data. Samples without RPKM and Raw read counts data were omitted. **(A)** Levels of mRNA expression among six genes. RPKM data from 296 samples were used, and the p-values were calculated using the Kruskal-Wallis test. **(B)** Levels of mRNA expression among different stages of HGSOC: Stage I (n = 0), Stage II (n = 18), Stage III (n = 239), Stage IV (n = 36). The *p*-value was calculated using the DESeq2 package. **(C)** Levels of mRNA expression among different grades of HGSOC: G1 (n = 1), G2 (n = 33), G3 (n = 254), G4 (n = 1). The *p*-value was calculated using the DESeq2 package. Each point represents an individual sample.

First, we analysed the expression levels of six key genes in the hippo pathway (*YAP1, TAZ, TEAD3, TEAD4, VGLL3*, and *VGLL4*) in HGSOC. We found that the expressions of *VGLL4*, *TEAD3*, *TEAD4*, and *YAP1* were all increased in HGSOC. By contrast, the expressions of *VGLL3* and *TAZ* were low in HGSOC (**Figure 1A**). The result was comparable to those obtained from the GSE9891 cohort (**Supplementary Figure S1A**). Interestingly, *VGLL3* expression was the lowest among all the genes in both the TCGA (*p=*<2e-16) and GSE9891 cohorts (*p=*<2e-16) (**Figure 1A** and **Supplementary Figure S1A**). When tumors were compared to normal ovarian samples, we found that *VGLL3* was significantly lower in tumor samples than it was in normal ovarian samples in both the TCGA and GSE26712 cohorts (*p*<0.05 and *p=*2.4e-07, respectively) (**Supplementary Figures S1B, C**). Whereas, *TEAD4* was significantly higher in tumor samples compared to normal ovarian samples in both the TCGA and GSE26712 cohorts (*p*<0.05 *p=*5.8e-07) (**Supplementary Figures S1B, C**). There were no significant differences observed regarding the expressions of *YAP1* and *VGLL4* between tumor and normal ovaries in either cohort. However, the expression of *TAZ* and *TEAD3* were discrepant between different datasets. Expression of *TAZ* in HGSOC was significantly lower in TCGA cohort (p<0.05), but higher in the GSE26712 cohort (*p=*2e-06) compared to normal ovary (**Supplementary Figures S1B, C**). No significant difference was observed in *TEAD3* expression between HGSOC *vs*. normal ovary in the TCGA cohort although it significantly increased in the GSE26712 cohort *p=*2.6e-05.

Finally, we checked the expression of VGLL3 both in mRNA and protein level in different OC cell lines. We observed different cell lines express different level of VGLL3 mRNA and protein (data not shown). Interestingly, we noticed that there was discrepancy of VGLL3 expression between mRNA and protein level in same cell line. The discrepancy was more prominent in SKOV3, YDOV13, and YDOV139 cell line (data not shown) and less prominent in OVCAR3 cell line, whereas, there was no observable discrepancy found in OVCA429, OVCA433, YDOV151, YDOV157 and YDOV161 cell lines in terms of VGLL3 expression at the mRNA and protein level.

Next, we explored the correlation of the above six hippo-related genes with the progression of HGSOC. *VGLL3* expression was found to be significantly increased in the advanced stages (stage III+IV) of HGSOC tumors (*p=*0.046) compared to in the early stage II (**Figure 1B**). However, *VGLL4, TEAD3, TEAD4, YAP*, and *TAZ* did not show any significant difference between stage II *vs*. stage III+IV in HGSOC (**Figure 1B**). When we checked the expressions of those six genes in different grades, we didn’t observe significant differences in any genes between (G1+G2) *vs*. (G3+G4). However, for *VGLL3* expression, there was a decreasing trend observed in (G3+G4) relative to (G1+G2) (*p=*0.098) (**Figure 1C**).

**Figure 2:**
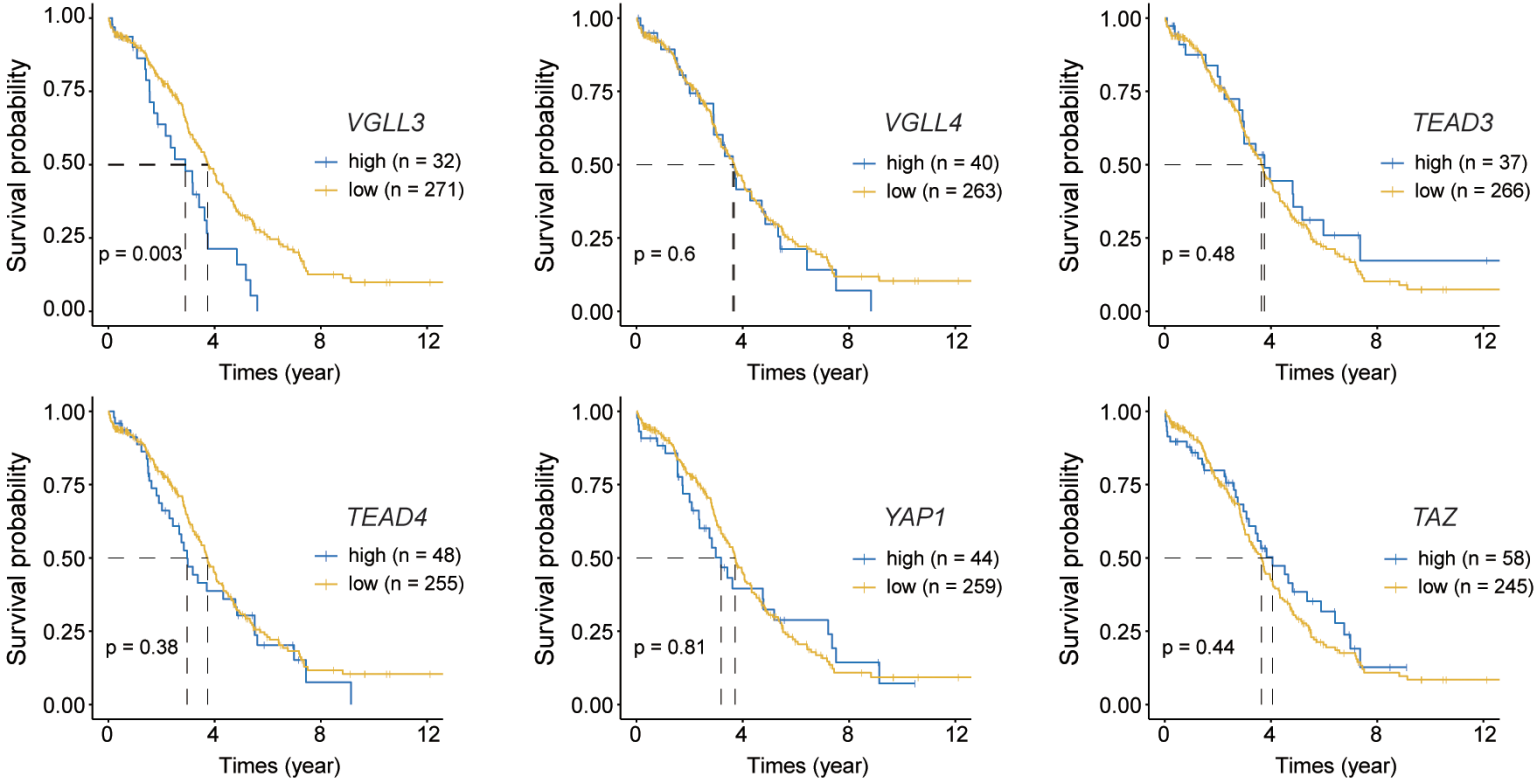
Kaplan–Meier curves for overall survival of six target genes. Normalised read counts data for 302 The Cancer Genome Atlas (TCGA) ovarian cancer samples were analysed for overall survival while comparing the high and low expression of each gene; Top: *from left to* right *VGLL3* (*p=*0.003), *VGLL4* (*p=*0.6), *TEAD3* (*p=*0.48); Bottom: *from left to right TEAD4* (*p=*0.38), *YAP1* (*p=*0.81), *TAZ* (*p=*0.44). High and low expression of each gene was selected based on the visual distinction described in **Supplementary Figure S2**. Statistical significance was evaluated using the log-rank test.

Next, we checked the correlations of those six genes with the OS of patients with HGSOC. The mRNA expression of each gene was categorised into high and low groups (**Supplementary Figure S2**). The data showed that only *VGLL3* had a significant association with OS (*p=*0.003) (**Figure 2**), which was consistent in both the GSE26712 and GSE9891 (*p=*0.048, *p=*0.012, respectively) cohorts (**Supplementary Figure S2B**, C *right panel*). *VGLL3*high correlated with the lower OS in HGSOC, while *VGLL3*low was associated with better OS. However, the other five genes did not show any significant correlation with OS (**Figure 2**). Taken together, these findings suggest that the role of *VGLL3* mRNA is distinctive in HGSOC compared to the other five genes, which correlated with the characteristics of advanced tumor stages of HGSOC and poor survival outcomes.

### *VGLL3* mRNA expression was associated with macrophage infiltration and unfavourable prognosis

**Figure 3:**
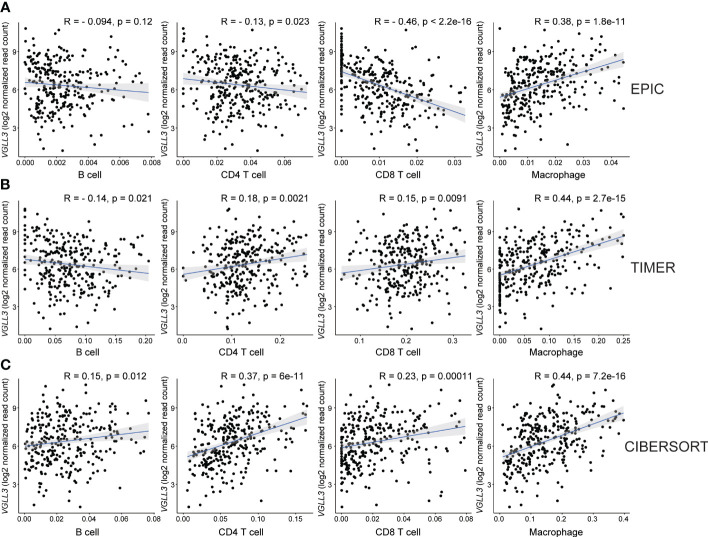
Correlation between *VGLL3* mRNA expression and immune cell infiltration. The levels of immune cell infiltrations were estimated using three databases: **(A)** EPIC, **(B)** TIMER, and **(C)** CIBERSORT abs mode. The three methods show that macrophage and *VGLL3* reached a consensus on a significantly positive correlation. The X-axis is the estimated values of three algorithms that represent immune cell fractions and the Y-axis represents the *VGLL3* mRNA expression. For CIBERSORT, cell fractions for each immune cell were considered as a summation of their subsets. After eliminating outliers of immune cell fractions using Tukey’s method, Pearson’s method was performed to determine the correlation between *VGLL3* gene and the immune cells (*from left to right*: B cell, CD4+ T cell, CD8+ T cell and macrophage), and the correlation coefficient was shown as R. For 303 The Cancer Genome Atlas (TCGA) ovarian cancer samples, the normalised read counts from TCGA Genome Data Analysis Center (GDAC) database and the immune cell infiltration levels from TIMER 2.0 website were downloaded and used to draw plots. A *p <*0.05 and R ≥ 0.3 was considered as statistically significant.

Then, we checked the correlation of six genes with immune cell infiltration and found that *VGLL3* was positively correlated with infiltration of macrophage, CD4+ T cell, and CD8+ T cell in HGSOC, while it was negatively correlated with B cells (**Figures 3A–C**). Interestingly, the correlation of *VGLL3* with macrophage infiltration was strongest among all other TIICs in HGSOC. This finding was consistently revealed in all three computational tools (EPIC, R=0.38; *p=*1.8e-11, TIMER, R=0.44; *p=*2.7e-15, and CIBERSORT, R=0.44; *p=*7.2e-16). However, we could not find any significant correlation between the other five genes and HGSOC TIICs (**Supplementary Figure S3**). Then, we checked the correlation of *VGLL3* with two different subtypes of macrophage (M1 and M2 macrophage). We noted that, while there was no significant correlation found between *VGLL3* with M1 macrophage (**Supplementary Figure S4A**), *VGLL3* was found to be significantly correlated with M2 macrophage infiltration R=0.44; *p=*5.7e-16 (**Supplementary Figure S4B**), thus suggesting that the association of *VGLL3* with macrophage mainly comes from M2 macrophage.

**Figure 4:**
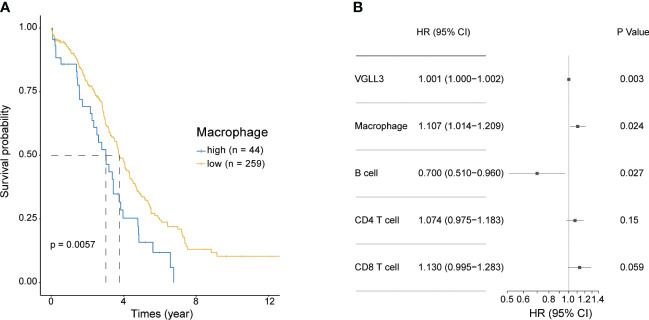
*VGLL3* and macrophages are unfavourable prognostic markers. Normalised read counts and infiltrated immune cell fractions estimated using EPIC for 303 HGSOC tissue samples were utilised. **(A)** Kaplan–Meier survival curves of OS comparing high (n=44) and low (n=259) macrophage infiltration in HGSOC, *p=*0.0057. **(B)** Forest plot visualizing the hazard ratio with a 95% confidence interval and *p*-value was calculated using multivariate Cox regression analysis. The levels of infiltrated immune cells estimated using EPIC were multiplied by 100 to transform them into percentile values. All variables were considered to be continuous variables.

Next, we investigated the association of macrophage infiltration with OS in HGSOC. Similar to VGLL3, high levels of macrophages were significantly correlated with poor overall survival (*p=*0.0057), whereas low levels of macrophage expression correlated with better OS (**Figure 4A**). Multivariate analysis further confirmed that *VGLL3* and macrophages were independent prognostic factors for HGSOC (*p=*0.003 and *p=*0.024, respectively) (**Figure 4B** and **Supplementary Figure S5**), thus suggesting that *VGLL3* serves as an independent unfavourable prognostic marker in HGSOC, possibly in association with macrophage infiltration.

### VGLL3 protein expression had similar effects on clinical outcomes as the *VGLL3* mRNA expression

**Figure 5:**
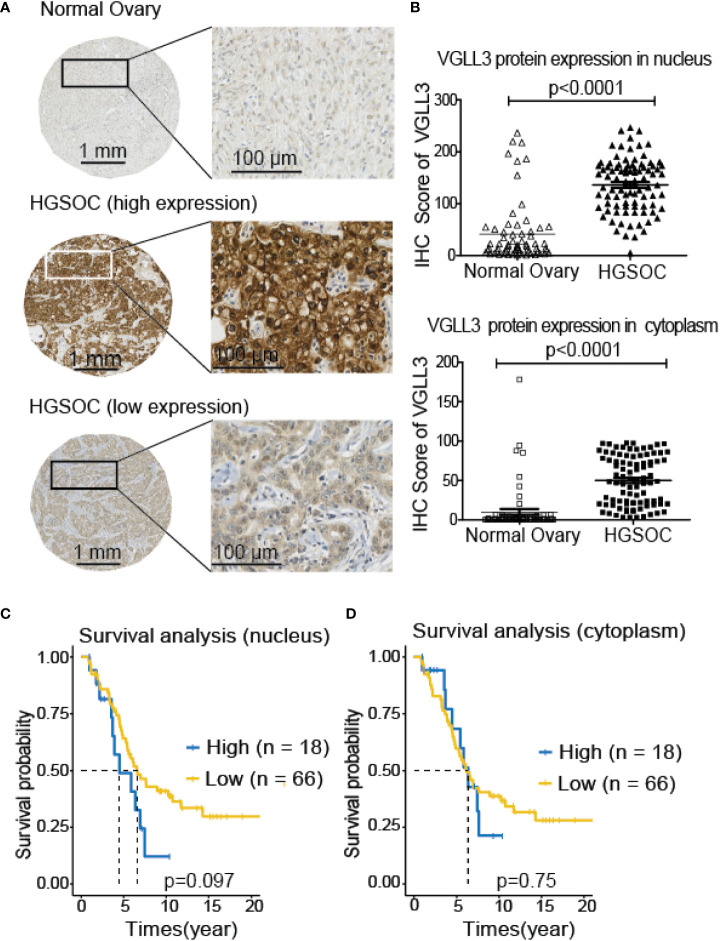
Expression of VGLL3 protein in normal ovary and HGSOC tissues and survival analysis depending on the expression levels of VGLL3 on nucleus and cytoplasm of cells. The unstained TMA blocks from 84 HGSOC and 66 adjacent normal ovarian tissues were immunohistochemically stained with anti-VGLL3 antibody, **(A)** Representative image of VGLL3 expression between HGSOC and adjacent normal ovary tissues. **(B)** IHC scores of VGLL3 in both nucleus and cytoplasm were calculated and compared between HGSOC and adjacent normal ovary, *p <*0.0001. **(C, D)** Kaplan–Meier survival curves of OS comparing high (n=18) and low (n=66) expression of VGLL3 protein in HGSOC are shown both in nucleus (*p=*0.097) and cytoplasm (*p=*0.75). Each point represents an individual sample.

Next, to investigate the significance of VGLL3 protein expression in HGSOC, immunohistochemistry-guided TMA score was analyzed at both nuclear and cytoplasmic level in our HGSOC cohort. Interestingly, VGLL3 protein expressions at both nuclear and cytoplasmic levels were significantly higher (*p*<0.001) in HGSOC tissues than it was in the normal adjacent tissues (**Table 2** and **Figures 5A, B**). Then, we investigated the correlation of VGLL3 protein expression at both nuclear and cytoplasmic levels with different clinicopathological features of HGSOC (**Table 3**). There was no significant difference found regarding the expression of VGLL3 protein depending on age, FIGO stage, tumor grade, Pre-CA125 level, and chemo-sensitivity (**Table 3**).

**Table 2:** Comparison of VGLL3 protein expression in high-grade serous ovarian carcinoma and normal adjacent tissues.

Variables	No	(%)	VgLL3_Nucleus		VgLL3_Cytoplasm	
			Mean IHC score (95% CI)	*p-*value	Mean IHC score (95% CI)	*p-*value
Normal	66	44.0	41.07 [27.69-54.45]	<0.0001	9.94 [2.61-17.28]	<0.0001
HGSOC	84	56.0	133.28 [121.42145.14]		48.07 [41.56-54.58]	

A non-parametric Mann-Whitney U test was performed to compare each diagnosis parameter.

**Table 3:** VGLL3 protein expression in high-grade serous ovarian carcinoma according to the clinicopathological characteristics.

Variables	No	(%)	VgLL3_Nucleus		VgLL3_Cytoplasm	
			Mean IHC score (95% CI)	*p-*value	Mean IHC score (95% CI)	*p-*value
Age				0.336		0.502
≤50	35	42.0	125.55 [108.31-142.79]		44.70 [34.29-55.12]	
>50	49	58.3	138.8 [124.23_153.37]		50.47 [41.67-59.27]	
FIGO stage				0.148		0.063
I/II	13	15.5	153.65 [125.48-181.82]		62.56 [45.65-79.48]	
III/IV	71	84.5	128.97 [116.83-141.11]		45.32 [38.03-52.61]	
Grade				0.250		0.310
G1+G2	40	47.6	126.92 [110.78-143.07]		44.55 [34.83-54.28]	
G3	44	52.4	139.05 [123.66-154.45]		51.27 [41.99-60.54]	
Pre CA-125 level				0.487		0.331
Negative (≤35U/ml)	7	8.3	146.08 [107.32-184.83]		60.83 [37.63-84.02]	
Positive (>35U/ml)	77	91.7	132.11 [120.43-143.80]		46.91 [39.92-53.91]	
Chemosensitivity				0.270		0.331
Sensitive	67	79.8	137.60 [125.19-150.00]		50.88 [43.47-58.29]	
Resistant	15	17.9	122.17 [95.95-148.38]		41.29 [25.63-56.96]	

A non-parametric Mann-Whitney U test was performed to compare each diagnosis parameter.

To evaluate the prognostic role of VGLL3 protein in HGSOC, we applied Kaplan–Meier survival analysis by determining the VGLL3^high^ and VGLL3^low^ groups in a manner similar to that of the mRNA data. We found that high expressions of VGLL3 protein at nuclear levels was correlated with lower OS in patients with HGSOC, although it was not statistically significant (**Figures 5C, D**).

### DEGs and pathway analysis identified altered pathways associated with *VGLL3*

**Figure 6:**
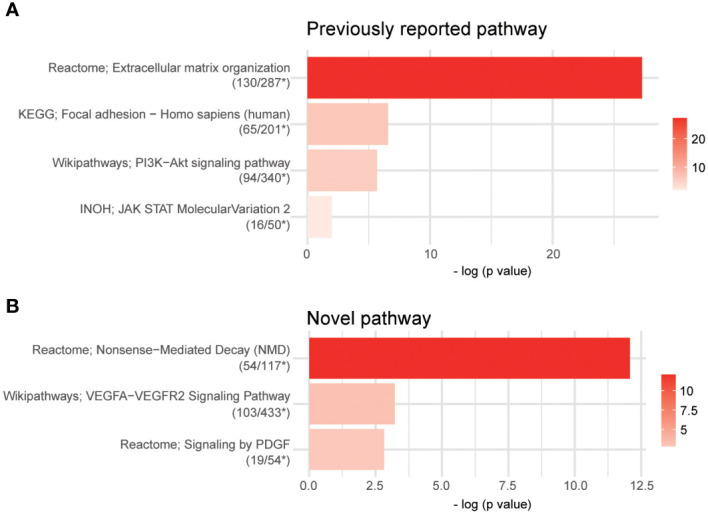
Pathway analysis using over-representation analysis from ConsensusPathDB (CPDB). Overall, 3,981 differentially expressed genes (DEGs) were identified after false discovery rate control (adjust *p*-value < 0.001, *p*-value < 2.04e-4). The cutoff p-value and *q*-value were 0.01 and 0.2 in overrepresentation analysis, respectively. **(A)** Four previously reported pathways and **(B)** three novel pathways were captured. Raw read counts for 296 samples from The Cancer Genome Atlas (TCGA) Genome Data Analysis Center (GDAC) database were used for DEGs analysis; *, The number of DEGs overlapped in the pathway/The total number of genes of the pathway.

We investigated VGLL3-related pathways in cancer to explore the potential mechanism in HGSOC. The DEG analysis showed that around 3,981 genes were significantly associated with *VGLL3* expression. Pathway analysis using 3,981 DEGs revealed that the gene sets associated with *VGLL3* mRNA expression showed enhancements in extracellular matrix organization, focal adhesion, PI3K-Akt signalling pathway, and JAK-STAT signalling pathway (**Figure 6A**). The association of *VGLL3* with those pathways has been previously been reported in different cancers (21). Interestingly, along with previously reported pathways, we identified three novel pathways that were associated with *VGLL3*: Nonsense-Mediated Decay pathways (NMD), vascular endothelial growth factors A-vascular endothelial growth factor receptor 2 (VEGFA-VEGFR2) signalling pathway, and platelet-derived growth factor (PDGF) signalling pathway (**Figure 6B**). The heatmap of DEGs revealed a strong correlation with high *vs*. low *VGLL3* mRNA expressions (**Supplementary Figure S6**).

## Discussion

In recent years, the role played by VGLL3 in cancers has attracted increasing research attention because of its dynamic behaviour in different cancers (18–22). In this study, we found that VGLL3 was an independent unfavourable prognostic factor of HGSOC, likely by affecting immune cells infiltration, particularly macrophages, and regulating or deregulating a significant number of oncogenic genes and pathways. The results suggest that VGLL3 is an important factor for HGSOC that correlates with tumor progression and immune evasion.

The malignancy of EOC largely depends on the constitutive activation/deactivation of different oncogenes, tumor suppressor genes, and transcription factors (40, 41). The correlation of VGLL3 in cancer proliferation, advanced tumor stage, grade, and poor prognosis has previously been reported in other cancers except for ovarian cancer (20–22). In line with previous reports, we found that VGLL3 expression in both mRNA and protein level was also correlated with advanced tumor stage and poor prognosis in HGSOC, suggesting that VGLL3 may promote the progression of HGSOC. Importantly, we may suggest a distinctive role of VGLL3 that conflicts with the previous report by Gambaro et al. where VGLL3 induction showed a tumor-suppressive phenotype in EOC (22). Given the ambivalent nature of genes with both oncogenic and tumor suppressor features, such as SMAD3 (42, 43), the association of VGLL3 with a significant number of genes in the DEG analysis suggests that the role of VGLL3 is too complex to be defined in a simple way. Therefore, a separate study using other OC cell lines is needed to reveal the mechanism of VGLL3 in EOC more precisely.

In contrast to the reduced expression of *VGLL3* mRNA in HGSOC, we found that VGLL3 protein expression was increased in HGSOC in comparison with adjacent normal ovarian tissue. A decent explanation for this discrepancy is currently unknown; one possible hypothesis might involve the effect of post-transcriptional modification. For example, mRNA regulatory elements and the affinity of RNA Binding Proteins (RBDs) increase RNA stability and translational efficiency of mRNA molecules, which leads to the aberrant expression of protein in tumor cells (44, 45). Therefore, we assume that post-transcriptional modifications, such as of *VGLL3* mRNA regulatory element and RBD, are more active in HGSOC than in the normal, which may increase VGLL3 mRNA stability as well as protein translation, and thereby increase its overexpression in protein level. Alternatively, the post-translation modification of VGLL3 protein might also play a role in stabilizing the VGLL3 protein, preventing their degradation, and thus increasing VGLL3 protein in HGSOC tumors. There is a need for an in-depth study to explore the post-transcriptional regulation of VGLL3 and its effect on VGLL3 mRNA stability and protein expression. Despite the fact that there is a discrepancy in VGLL3 mRNA and protein expression between tumor and normal tissues, we interestingly observed that higher levels of both VGLL3 mRNA and protein expression among tumor tissues were associated with the worse OS in HGSOC.

Tumor-infiltrating lymphocytes and the immune status of the tumor microenvironment have been reported to affect progression, therapeutic effects, and recurrence in many cancers (46). Tumor-associated macrophages (TAMs), which mainly belong to the M2 macrophage phenotype, are known to correlate with poor outcomes in solid cancers and play important roles in tumorigenesis (47). A high density of CD163+ M2-macrophages is predominantly associated with poor prognosis in ovarian cancer and known to be involved in tumor invasion, angiogenesis, metastasis, and early recurrence (48, 49). Moreover, the high M1/M2 ratio of tumor infiltrating macrophages correlates with prolonged survival time in EOC, while low M1/M2 ratio correlates with poor OS (50, 51). In this study, we also observed the association of *VGLL3* with macrophage infiltration in HGSOC, which likely contributed to the worsening of OS. Moreover, we found that M2 macrophage was more strongly correlated with *VGLL3* than M1 macrophage, suggesting that VGLL3 may be involved in the poor prognosis of HGSOC by association with macrophages, particularly M2 macrophage.

In addition to known clinical and molecular biomarkers such as *TP53*, *BRCA1/2*, and *MYC*, *VGLL3* regulates many key genes and pathways, and it is also related to clinical prognosis. High *VGLL3* expression has been found to activate several signalling pathways, such as MAPK, JAK-STAT, PI3K/Akt/mTOR, ECM, focal adhesion, and WNT pathways in many tumors (20, 21). In this report, we also discovered those pathways in HGSOC that correlated with high *VGLL3* expression. Further, we found three novel pathways (NMD, VEGFA-VEGFR2, PDGF) that were associated with high *VGLL3*, suggesting that *VGLL3* may regulate key genes in those pathways. Frequent activation of ECM, PI3K/Akt/mTOR, and VEGFA-VEGFR2 signalling pathways has been associated with higher invasive and migratory capacities in subpopulations of human OC (52–54). On the other hand, TAMs have reported to correlate with many signalling pathways including PI3K/AKT/mTOR signalling pathway, ECM, and focal adhesion molecules that modulate the tumor microenvironment (55–57). In this study, we connected *VGLL3* with macrophage infiltration and signalling pathways and demonstrated that VGLL3 may play a critical role in the poor prognosis of HGSOC. There is still a need for further research to show the direct interaction and functional interplay of VGLL3 with related molecules. It is important to identify new biomarkers to improve the management of ovarian cancer patients. In particular, focus should be given on non-invasive characterization of cancer to discover more efficient markers. Radiomics analysis using imaging techniques and implementations of new radiopharmaceuticals based on molecular features of tumor cells would be a good tool for non-invasive staging, prognosis and restaging of cancers. 2-[^18^F] FDG PET/CT is considered the most popular and useful imaging technique for relapse detection in ovarian cancer patients, offering prognostic value. Ongoing research is conducting to explore new biomarkers in ovarian cancer patients by analysing imaging biomarkers in combination with clinical, pathological, and analytical data (58, 59).

Obviously, the limitation of our study is that it used public mRNA data not derived from our own patients and lacked evidence from *in vitro* and *in vivo* research. Nonetheless, the public DBs including TCGA contain decent numbers of curated data, and the findings were highly correlated with our immunohistochemistry-guided TMA in HGSOC tissue samples. Therefore, VGLL3 has been strongly suggested to have significance and a potential role in both mRNA and protein levels, which represents the strength of this study. Currently, we are conducting research into investigating the molecular mechanism of VGLL3 in HGSOC as a continuation of this study.

## Conclusion

This study demonstrated that VGLL3 has potential prognostic value in HGSOC because its overexpression was shown to be associated with advanced tumor stage and poor prognosis. Moreover, VGLL3 was associated with TAM infiltration, which is frequently observed in the immunosuppressive microenvironment of cancer. Finally, the DEGs pathways and co-expressing genes identified in this study suggest the prospective molecular function of VGLL3 in cancer.

## Data availability statement

The original contributions presented in the study are included in the article/**Supplementary Material**. Further inquiries can be directed to the corresponding authors.

## Ethics statement

The studies involving human participants were reviewed and approved by Institutional Review Board of Samsung Medical Center (IRB#, SMC 2021-06-23), Seoul, Republic of Korea, and Institutional Review Board of Gangnam Severance Hospital (IRB#, HTB-P2021-5), Seoul, Republic of Korea. The patients/participants provided their written informed consent to participate in this study.

## Author contributions

Conception and design: RH, JY and E-SK. Curation of the data: RH, JL and JY. Analysis and interpretation of data: RH, JL, JY and E-SK. Collection of tissue sample and clinical information: H-YS, HK and J-HK. TMA guided immunohistochemistry perform and analysis: J-YC. Writing, review and revision of the manuscript: RH, JL, JY and E-SK. Supervision and fund acquisition: E-SK. All authors contributed to the article and approved the submitted version.

## Supplementary material

Supplementary Figure 1 mRNA expression levels of six target genes in HGSOC samples. **(A)** Expression of six target genes of 264 ovarian cancer tissues. *p* value was calculated using Kruskal-Wallis test. **(B)** Expression of six target genes analysed by GEPIA2 in ovarian cancer (n=426) specimen and compared to normal ovarian tissue (n=88). Red color means ovarian cancer tissues and blue color means normal ovarian tissues; *, *p*<0.05 **(C)** Expression of six target genes of 185 ovarian cancer tissues compared to those of 10 normal ovarian tissues. *p* value was calculated using Mann-Whitney U test.

Supplementary Figure 2 Expression of six target genes and Kaplan-Meier plot. **(A)** The distribution of each gene expression among 303 samples of The Cancer Genome Atlas (TCGA) ovarian cancer. Dashed line represents the cut-off for high and low expression. **(B)** *Left*: Set the cut-off for high *VGLL3* and low *VGLL3* expression in GSE26712 dataset, *Right:* Kaplan–Meier survival curves of OS comparing high (n=37) and low (n=148) expression of *VGLL3* in HGSOC, *p=*0.048. **(C)** *Left*: Set the cut-off for high *VGLL3* and low *VGLL3* expression in GSE9891 dataset, *Right:* Kaplan–Meier survival curves of OS comparing high (n=93) and low (n=171) expression of *VGLL3* in HGSOC, *p=*0. 012. **(D)** Set the cut-off for high macrophage and low macrophage infiltrations in EPIC data. **(E)** Set the cut-off for high *VGLL3* and low *VGLL3* expression in TMA data.

Supplementary Figure 3 Correlation between five target genes (*VGLL4, TEAD3, TEAD4, YAP1*, and *TAZ*) and the level of infiltrated immune cells using **(A)** EPIC, **(B)** TIMER, and **(C)** CIBERSORT abs mode. The X-axis is the estimated values of three algorithms that represent immune cell fractions and the Y-axis represents the *VGLL3* mRNA. For CIBERSORT, cell fractions for each immune cell as a summation of their subsets were considered. After eliminating outliers using Tukey’s method, Pearson’s method was performed to find the correlation between *VGLL3* genes and the immune cells (from left to right: B cell, CD4+ T cell, CD8+ T cell and macrophage), and the correlation coefficient was shown as R. A *p*<0.05 was considered as statistically significant.

Supplementary Figure 4 Correlation of VGLL3 with the subsets of macrophages. After eliminating outliers of the level of infiltrated immune cells using Tukey’s method, Pearson’s method was performed to find the correlation between VGLL3 gene and **(A)** M1 macrophage and **(B)** M2 macrophage. Correlation coefficient was shown as R. A p<0.05 and R≥ 0.30 was considered as statistically significant.

Supplementary Figure 5 Forest plot visualizing hazard ratios with 95% confidence interval and p-values calculated using multivariate Cox regression analysis. Levels of infiltrated immune cells were estimated using TIMER. All variables were considered continuous variables.

Supplementary Figure 6 Heatmap of DEGs between the *VGLL3* ^high^ and *VGLL3* ^low^ group. For the heatmap, the z score normalised value from the results of DESeq2 were used. The genes presented in the heatmap were selected genes that overlapped with the cancer gene list provided by Bushman laboratory (See Methods). Previously reported pathways had 128 overlapped genes in total, and novel pathways had 55 overlapped genes in total. * previously reported pathways; ** newly found pathways in this study.
