# Supplementary figures and images for: VGLL3 expression is associated with macrophage infiltration and predicts poor prognosis in epithelial ovarian cancer
Source: Front Oncol. 2023 Jun 5;13:1152991. doi: 10.3389/fonc.2023.1152991 (PMC10277618; doi:10.3389/fonc.2023.1152991)

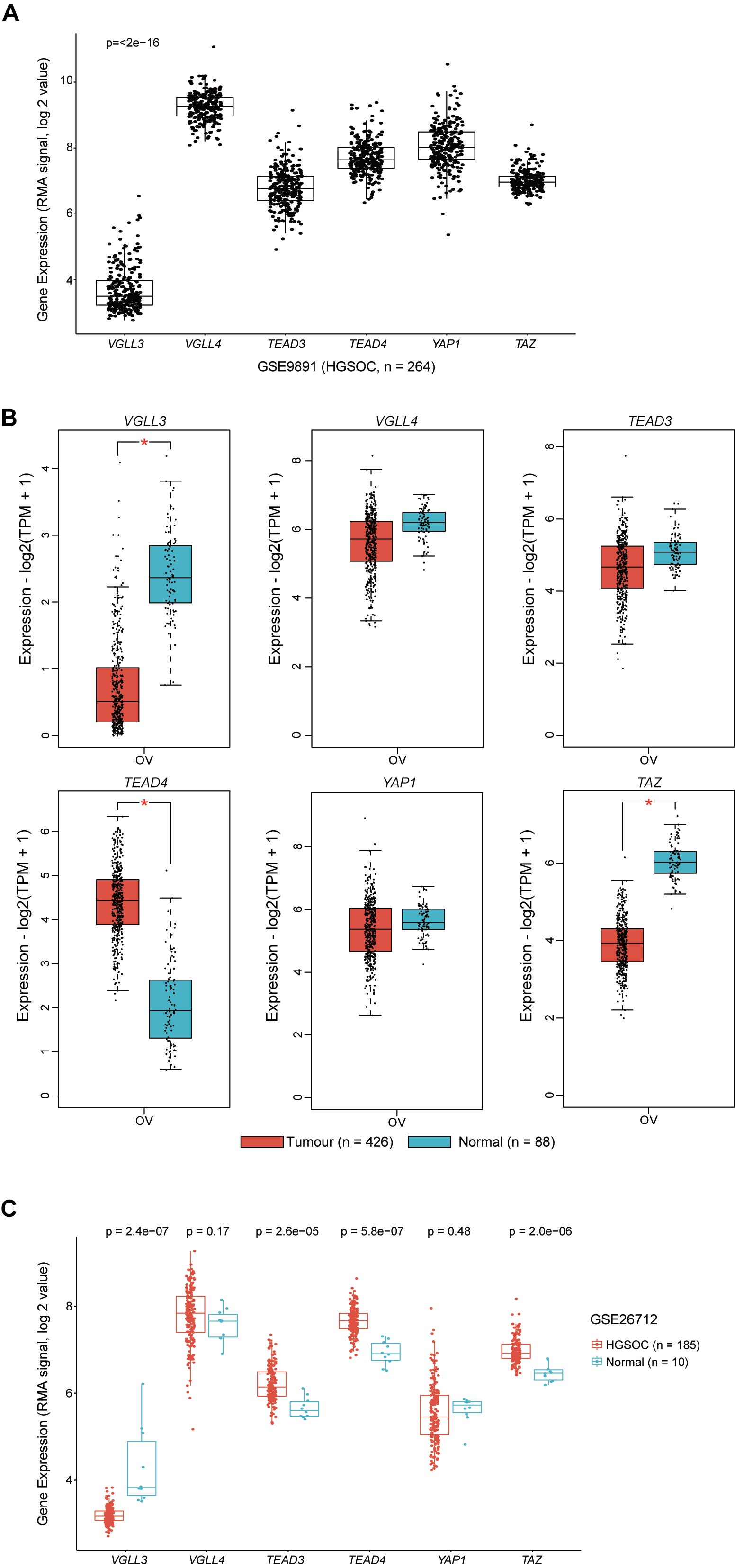

Supplement: Supplementary Figure 1 — mRNA expression levels of six target genes in HGSOC samples. (A) Expression of six target genes of 264 ovarian cancer tissues. p value was calculated using Kruskal-Wallis test. (B) Expression of six target genes analysed by GEPIA2 in ovarian cancer (n=426) specimen and compared to normal ovarian tissue (n=88). Red color means ovarian cancer tissues and blue color means normal ovarian tissues; *, p<0.05 (C) Expression of six target genes of 185 ovarian cancer tissues compared to those of 10 normal ovarian tissues. p value was calculated using Mann-Whitney U test. [file Image_1.jpg]

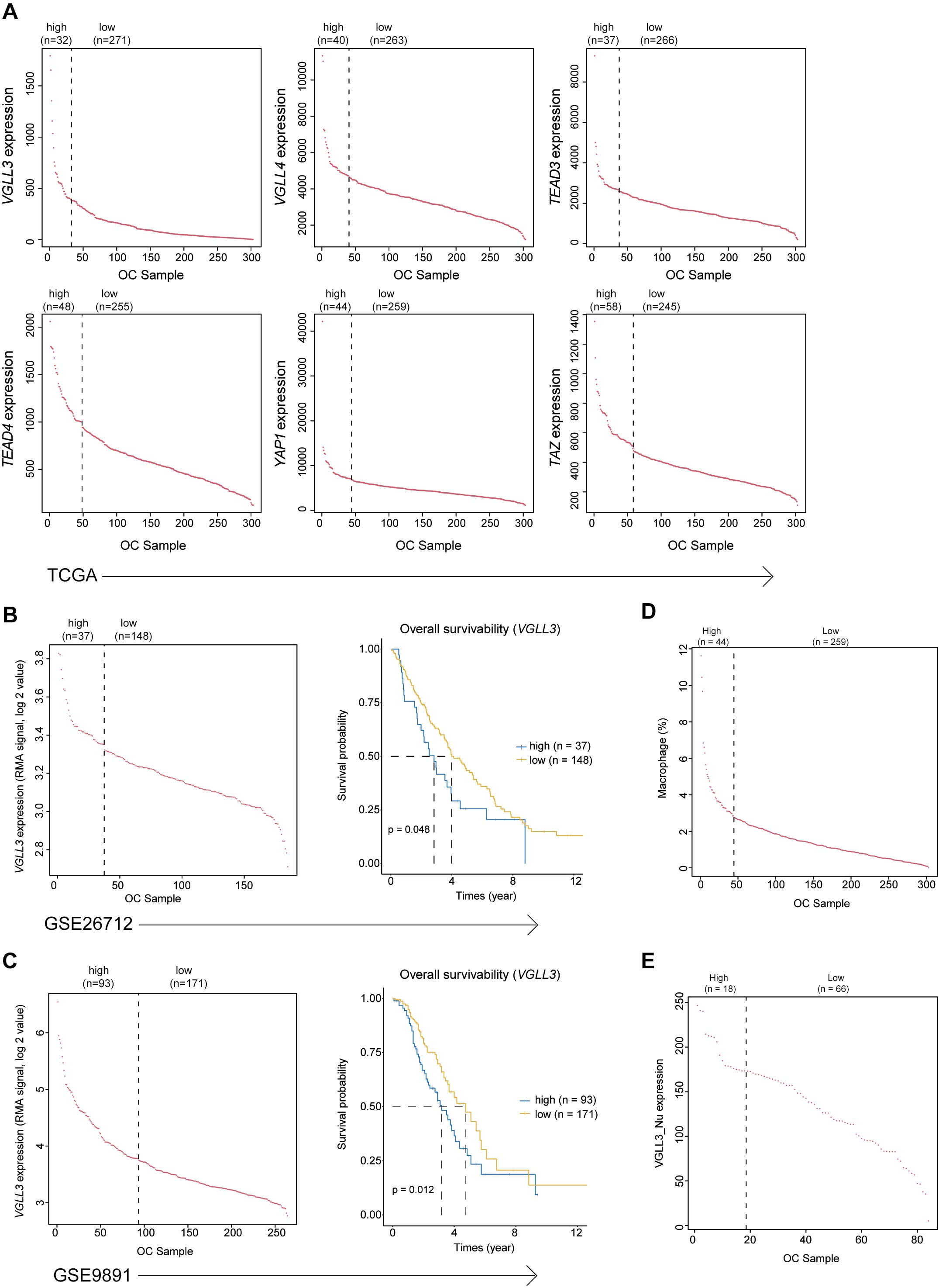

Supplement: Supplementary Figure 2 — Expression of six target genes and Kaplan-Meier plot. (A) The distribution of each gene expression among 303 samples of The Cancer Genome Atlas (TCGA) ovarian cancer. Dashed line represents the cut-off for high and low expression. (B) Left: Set the cut-off for high VGLL3 and low VGLL3 expression in GSE26712 dataset, Right: Kaplan–Meier survival curves of OS comparing high (n=37) and low (n=148) expression of VGLL3 in HGSOC, p=0.048. (C) Left: Set the cut-off for high VGLL3 and low VGLL3 expression in GSE9891 dataset, Right: Kaplan–Meier survival curves of OS comparing high (n=93) and low (n=171) expression of VGLL3 in HGSOC, p=0. 012. (D) Set the cut-off for high macrophage and low macrophage infiltrations in EPIC data. (E) Set the cut-off for high VGLL3 and low VGLL3 expression in TMA data. [file Image_2.jpg]

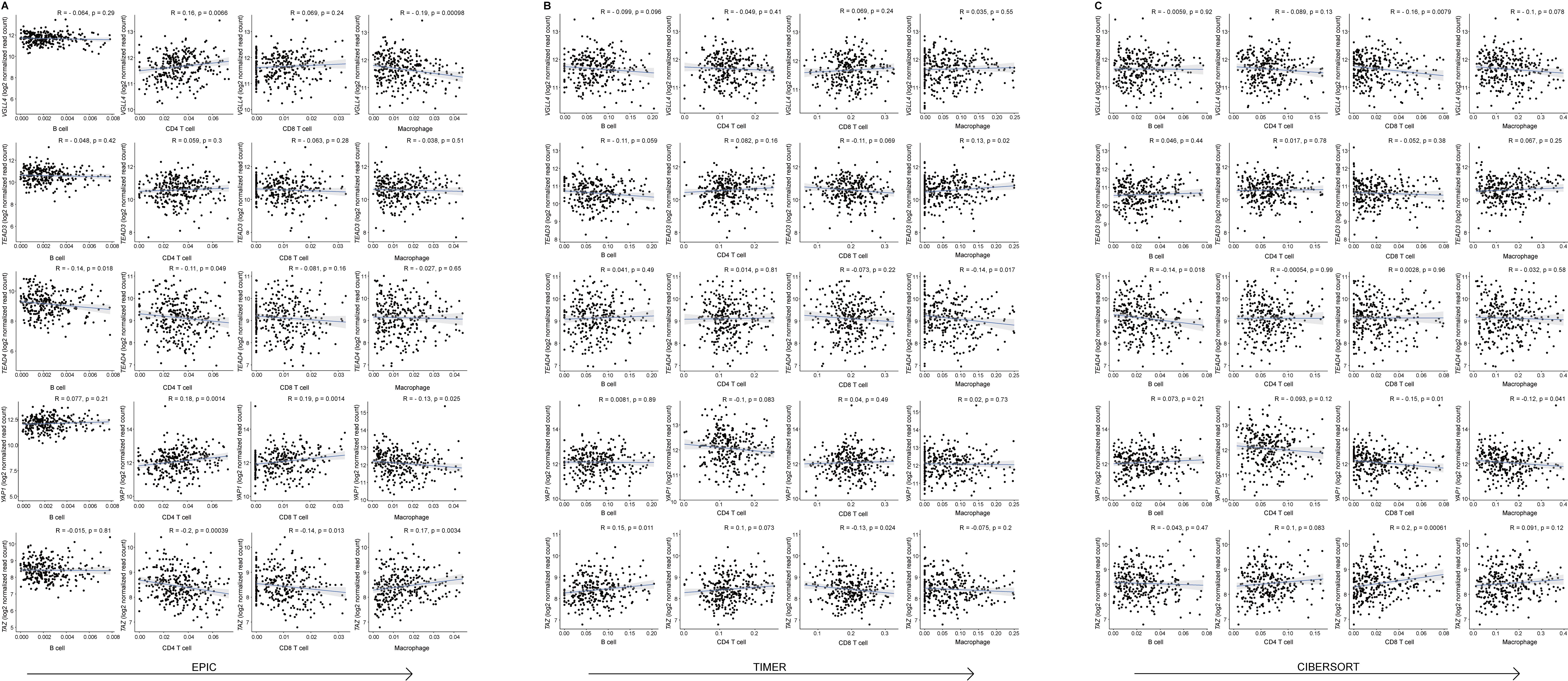

Supplement: Supplementary Figure 3 — Correlation between five target genes (VGLL4, TEAD3, TEAD4, YAP1, and TAZ) and the level of infiltrated immune cells using (A) EPIC, (B) TIMER, and (C) CIBERSORT abs mode. The X-axis is the estimated values of three algorithms that represent immune cell fractions and the Y-axis represents the VGLL3 mRNA. For CIBERSORT, cell fractions for each immune cell as a summation of their subsets were considered. After eliminating outliers using Tukey’s method, Pearson’s method was performed to find the correlation between VGLL3 genes and the immune cells (from left to right: B cell, CD4+ T cell, CD8+ T cell and macrophage), and the correlation coefficient was shown as R. A p<0.05 was considered as statistically significant. [file Image_3.jpg]

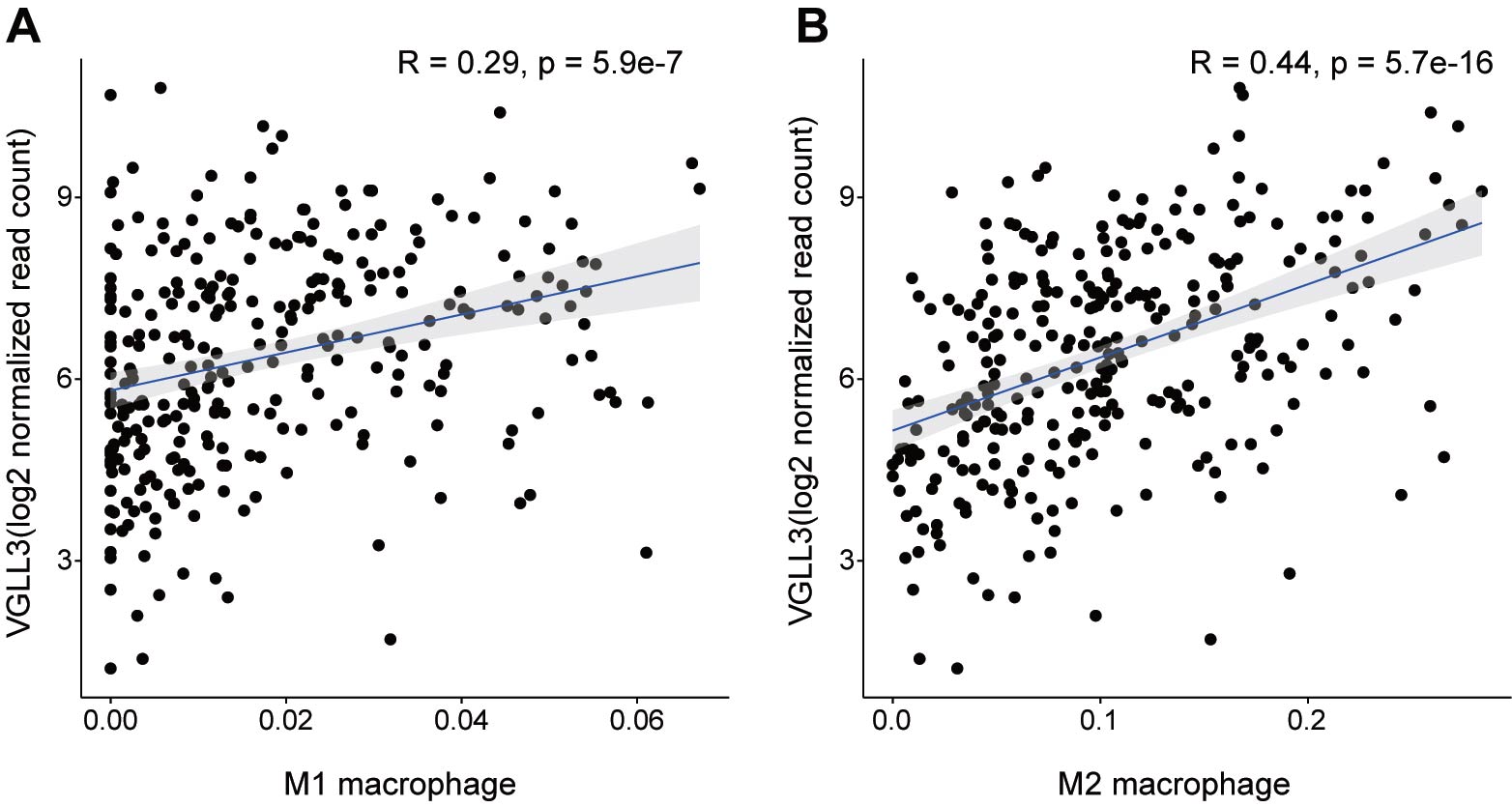

Supplement: Supplementary Figure 4 — Correlation of VGLL3 with the subsets of macrophages. After eliminating outliers of the level of infiltrated immune cells using Tukey’s method, Pearson’s method was performed to find the correlation between VGLL3 gene and (A) M1 macrophage and (B) M2 macrophage. Correlation coefficient was shown as R. A p<0.05 and R≥ 0.30 was considered as statistically significant. [file Image_4.jpg]

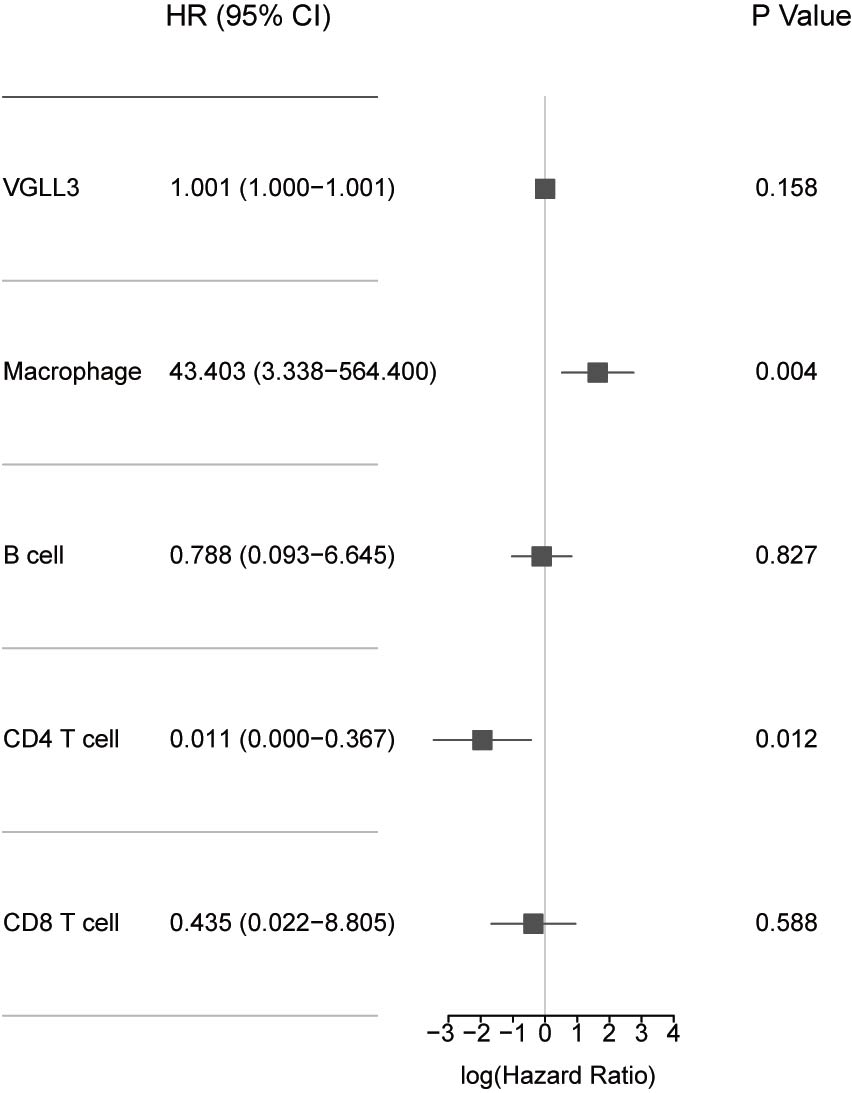

Supplement: Supplementary Figure 5 — Forest plot visualizing hazard ratios with 95% confidence interval and p-values calculated using multivariate Cox regression analysis. Levels of infiltrated immune cells were estimated using TIMER. All variables were considered continuous variables. [file Image_5.jpg]

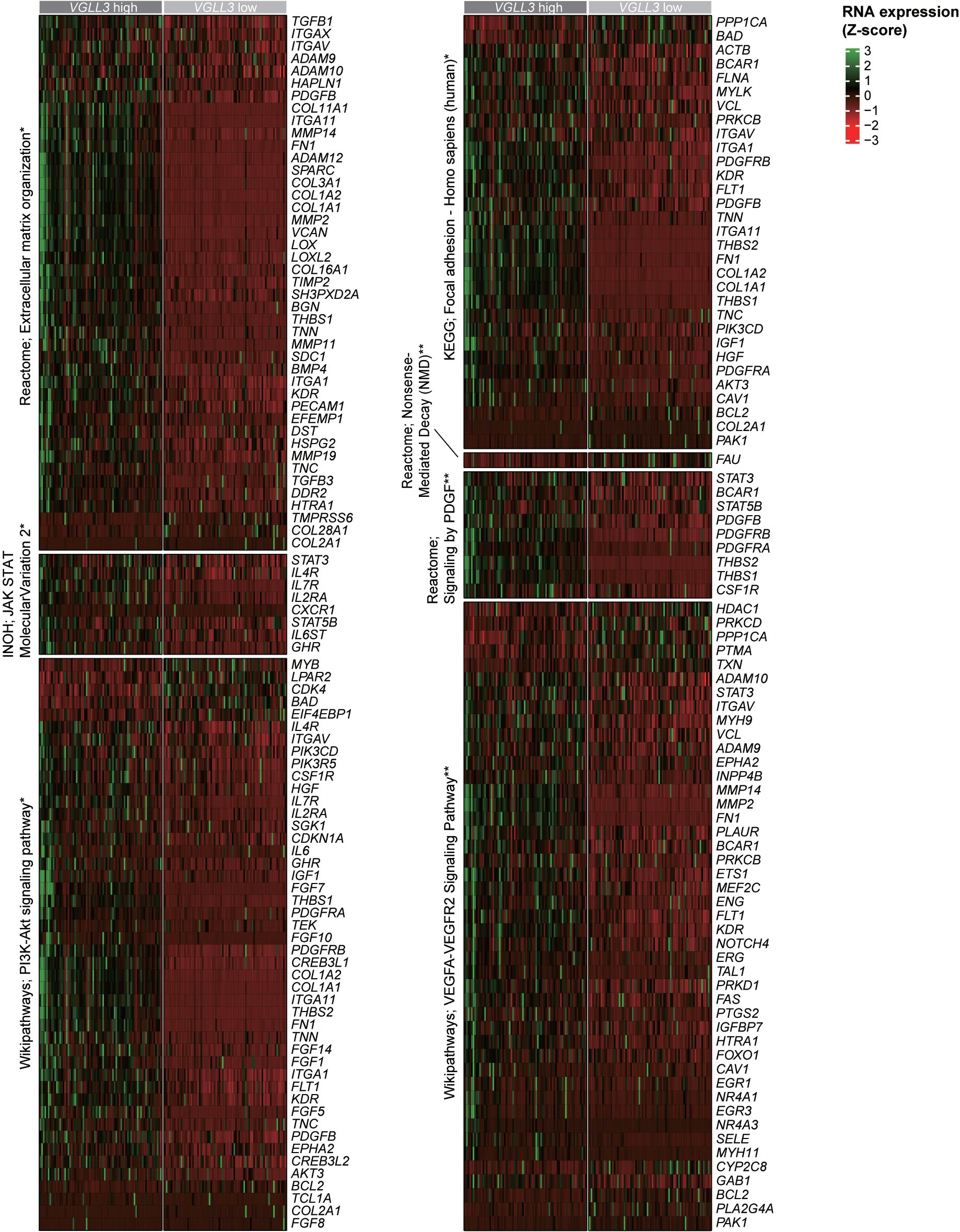

Supplement: Supplementary Figure 6 — Heatmap of DEGs between the VGLL3 high and VGLL3 low group. For the heatmap, the z score normalised value from the results of DESeq2 were used. The genes presented in the heatmap were selected genes that overlapped with the cancer gene list provided by Bushman laboratory (See Methods). Previously reported pathways had 128 overlapped genes in total, and novel pathways had 55 overlapped genes in total. * previously reported pathways; ** newly found pathways in this study. [file Image_6.jpg]
